# Serosal Patching with Glubran^®^2 on the Pancreatic Stump for Reducing Postoperative Pancreatic Fistulae After Robot-Assisted Distal Pancreatectomy: A Single-Center Retrospective Study

**DOI:** 10.3390/cancers17030502

**Published:** 2025-02-03

**Authors:** Ahmad Mahamid, Eden Gerszman, Esther Kazlow, Aasem Abu Shtaya, Natalia Goldberg, Dvir Froylich, Riad Haddad

**Affiliations:** 1 Divion of Surgery, Carmel Medical Center, Haifa 3436212, Israel; eden.gerszman@gmail.com (E.G.); ekazlow@gmail.com (E.K.); dvirfr7@gmail.com (D.F.); dr.riad.haddad@gmail.com (R.H.); 2 The Ruth and Bruce Rappaport Faculty of Medicine, Technion, Israel Institute of Technology, Haifa 3525433, Israel; asemab@clalit.org.il (A.A.S.); natalia.goldberg@gmail.com (N.G.); 3 Department of Gastroenterology, Carmel Medical Center, Haifa 3436212, Israel; 4 Department of Radiology, Carmel Medical Center, Haifa 3436212, Israel

**Keywords:** pancreatectomy, serosal patching, sealant, postoperative pancreatic fistula, Glubran^®^2, robot-assisted left pancreatectomy, postoperative complications, pancreatectomy

## Abstract

Background: Postoperative pancreatic fistulae (POPFs) are a significant cause of morbidity following left pancreatectomy. We hypothesized that incorporating serosal patching with the application of a synthetic sealant, a modified cyanoacrylate (Glubran^®^2), to the pancreatic stump, would decrease the incidence rate of clinically significant POPFs. Methods: This is a retrospective study of consecutive patients who underwent robot-assisted left pancreatectomy. The primary outcome was clinically significant POPFs within 90 days of surgery. Secondary outcomes included the incidence rate of POPFs (all the grades), 90-day morbidity, and 90-day mortality. Results: We compared outcomes between Glubran^®^2 sealant with serosal patching (GSP, *n* = 6) and Glubran^®^2 sealant without serosal patching (GNSP, *n* = 12) groups. The GSP group had significantly lower incidence rates of clinically significant POPFs (grades B/C) (*p* = 0.034) and overall POPFs (all the grades) (*p* = 0.046). No significant differences in 90-day postoperative morbidity were observed between the two groups (*p* = 0.56), and no 90-day mortality occurred in either group. Conclusions: Incorporating serosal patching along with Glubran^®^2 sealant in the management of the pancreatic stump during left pancreatectomy demonstrates promising results in reducing the incidence rate of clinically significant POPFs. This finding highlights the need for further research with larger sample sizes in order to confirm the observed outcomes and explore the long-term implications for postoperative complications and recovery in patients undergoing this procedure during pancreatic surgery.

## Simple Summary

Postoperative pancreatic fistulae (POPFs) are a significant cause of morbidity following left pancreatectomy. The aim of our retrospective study was to investigate whether adding serosal patching to Glubran^®^2 sealant application to the pancreatic stump during robot-assisted left pancreatectomy reduces postoperative pancreatic fistulae (POPFs). This study compared six patients receiving Glubran^®^2 with serosal patching (GSP) to twelve receiving Glubran^®^2 alone (GNSP). The GSP group showed a significantly lower incidence rate of clinically significant POPFs (grades B/C, *p* = 0.034) and overall POPFs (all the grades, *p* = 0.046). The ninety-day morbidity and mortality were similar between the groups. Although our results suggest that combining serosal patching with Glubran^®^2 sealant reduces POPFs, further research with a larger cohort is needed to confirm these findings and assess long-term impacts on recovery. Our study highlights the potential benefit of this combined approach in mitigating a significant complication of left pancreatectomy.

## 1. Introduction

Left pancreatectomy procedures performed at high-volume hepato-pancreato-biliary centers have shown low mortality rates but are associated with high incidence rates of perioperative and postoperative complications. Postoperative pancreatic fistulae (POPFs) emerge as the primary contributor to postoperative morbidity following pancreatic resections, impacting approximately 30–35% of cases [1,2,3]. POPFs present with a constellation of early complications, encompassing impaired gastric motility, the development of intra-abdominal suppurative collections, systemic inflammatory response syndrome (SIRS) progressing to sepsis, significant postoperative bleeding, and a heightened risk of mortality [4,5]. POPFs significantly increase healthcare expenditures by substantially prolonging hospitalizations, thereby almost doubling overall hospital costs [6]. Furthermore, patients experiencing POPFs exhibit unfavorable long-term outcomes, including increased susceptibility to cancer recurrence [7,8,9].

Glubran^®^2, a synthetic biodegradable adhesive comprising N-butyl-cyanoacrylate (NBCA) and metacrylosysolfolane (MS), was developed by GEM s.r.l. in Viareggio, Italy. This adhesive polymerizes rapidly upon contact with living tissues, forming a thin, elastic film with superior tensile strength for robust tissue adhesion. This film naturally conforms to the tissues to which it is applied and exhibits impermeability to liquids and resistance to adaptations induced by blood or organic body fluids [10].

Glubran^®^2 has proven to be effective in preventing leaks and reducing postoperative morbidity, when utilized as reinforcement in the stapler line during laparoscopic sleeve gastrectomy (LSG) [10]. It has also demonstrated a decrease in the rate of alveolar air leaks following pulmonary resections [11]. A 2019 study [12] reported Glubran^®^2’s efficacy in reducing leaks from the stapler line when combined with omentopexy in LSG.

However, the effectiveness of Glubran^®^2 in preventing POPFs has not been investigated to date. The use of Glubran^®^2 in serosal patching may be effective in preventing the leakage of gastrointestinal fluids caused by postoperative complications, such as POPFs. Therefore, we hypothesized that the application of Glubran^®^2 with serosal patching to the pancreatic stump following robot-assisted left pancreatectomy would decrease the incidence rate of clinically significant (Grades B/C) POPFs.

## 2. Materials and Methods

Patients who underwent robot-assisted left pancreatectomy between January 2023 and June 2024 were identified from the surgical databases at Carmel Medical Center (Haifa, Israel), a university-affiliated tertiary care hospital with a substantial hepato-biliary unit and over two decades of experience in advanced hepato-pancreato-biliary surgery. The data were collected from the Chameleon database, a comprehensive electronic medical record system, which includes detailed patient information and perioperative data. This study was conducted in accordance with the Declaration of Helsinki and Good Clinical Practice Guidelines and was approved by the Institutional Review Board (IRB) of Carmel Medical Center. Our review included the examination of patient demographics, detailed surgical histories, pathology results, and follow-ups from the medical/oncology team. All the patients received anesthesiologic clearance and multidisciplinary team surgical approval. All the patients were informed about the procedure, including risks and benefits. Written consent for surgery was obtained from all the patients. Because of the retrospective nature of this study, informed consent from patients was waived by the Institutional Review Board Committee.

### 2.1. Study Population

Patients aged 18–90 years who underwent robot-assisted left pancreatectomy were included in this study. Patients with local unresectable disease or intra-abdominal metastasis were excluded.

### 2.2. Operative Technique

All the patients underwent robot-assisted left pancreatectomy with splenectomy and lymphadenectomy, using the radical antegrade approach [13] and the Da Vinci XI surgical platform. The patients were put in a supine position with split legs at a 15° rightward tilt and a 30° reverse Trendelenburg position. Following laparoscopic exploration of the abdominal cavity, a total of four 8 mm robotic trocars were inserted in a horizontal line at the level of the umbilicus and 8 cm apart from each other. An additional 12 mm AirSeal assistant trocar was inserted at the level of the Pfannenstiel incision point. The 12 mm trocar was removed at the end of the operation, and the specimens were retrieved using a surgical bag through its incision. The incision was extended as needed. No additional ports were required. The wounds were then closed in layers after deflation of the pneumoperitoneum [14]. All the procedures were performed by a single surgical team (A.M. and R.H.).

### 2.3. Intervention

**Figure 1:**
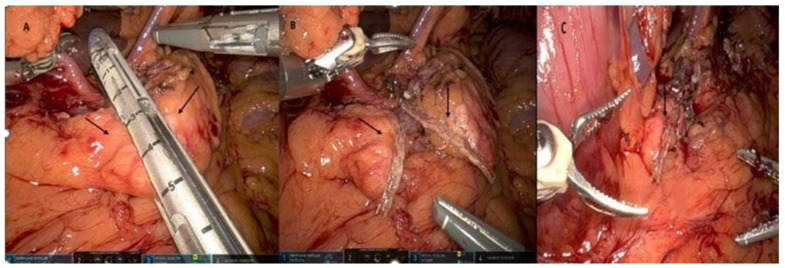
Transection of the pancreas. (**A**) Arrows show the pancreas before the transaction with the linear stapler; (**B**) arrows show the staple lines at the distal edge of the remaining pancreas and proximal edge of the resected specimen; (**C**) arrow shows the edge of the remaining pancreatic stump after the retrieval of the resected specimen.

**Figure 2:**
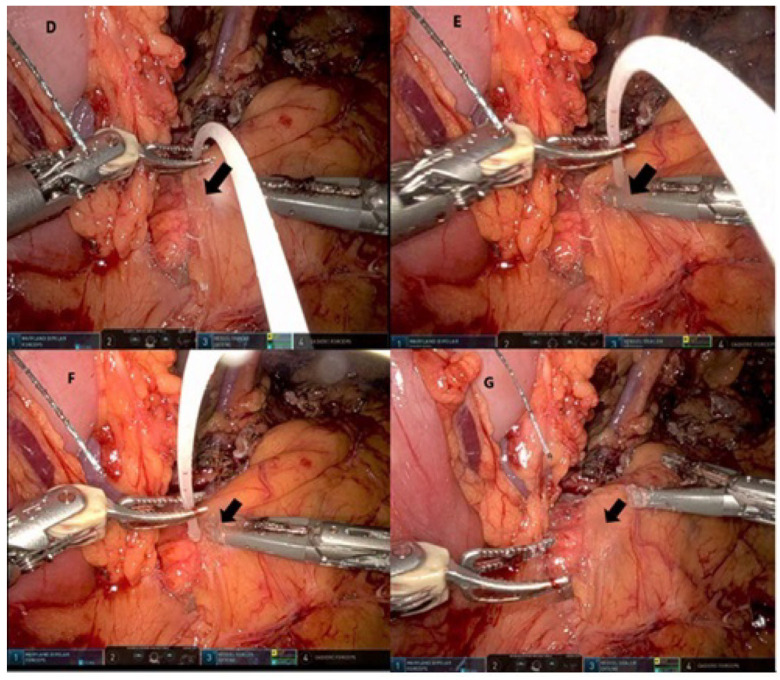
Covering the pancreatic stump with Glubran^®^2 sealant and serosal patching within the retroperitoneum. (**D**–**F**) Applying the Glubran^®^2 to the pancreatic stump while covering it with layers of retroperitoneal tissue, until achieving satisfactory adherence. (**G**) The final state of the pancreatic stump, satisfactorily covered under the retroperitoneum. Arrows indicate the area of the pancreatic stump.

In all the patients included in this study, the pancreas was transected using a linear stapler, with no farther ligation or suturing of Wirsung’s duct (Figure 1). Then, 2 mL of Glubran^®^2 sealant (GEM s.r.l., Italy) was applied to the pancreatic stump so that it overlapped the margins of the cut edge of the pancreas. In the group that received Glubran^®^2 sealant with serosal patching (GSP), following the application of the Glubran^®^2, layers of retroperitoneal tissue were used to cover the glued stumps, which were held in place until they achieved satisfactory adherence (Figure 2). In this group, the pancreatic stump was positioned in its original retroperitoneal anatomical place; then, the superior and inferior edges of the retroperitoneal tissue from around the resected pancreatic bed were used to cover the stump. An intraoperative drain was inserted at the surgical site near the pancreatic stump. The concentration of amylase in a sample of the drained fluid was measured and recorded on postoperative day 3.

### 2.4. Outcomes

This study’s primary objective was to determine the incidence rate of clinically significant POPFs. The International Study Group on Pancreatic Fistulae (ISGPF) classification system was employed, categorizing POPFs into biochemical (Grade A) and clinically significant (Grades B and C) fistulae.

Grade A POPFs are defined as possessing measurable fluid output with elevated amylase levels on postoperative day 3, lacking significant clinical consequences. Clinically significant POPFs (Grades B and C) are characterized as follows: Grade B necessitates at least one of the following: endoscopic or radiological intervention, drain retention exceeding three weeks, clinical-symptom-absent organ failure, or a clinically relevant alteration in management. Progression to Grade C occurs with the implementation of major management changes, deviation from standard clinical pathways, or the manifestation of organ failure [15]. Secondary outcome measures encompassed the overall incidence rate of POPFs (all the ISGPF grades), 90-day postoperative complications (assessed using the Clavien–Dindo classification) [16], 90-day mortality rate, the need for subsequent operative or procedural interventions, and the duration of postoperative hospitalization.

### 2.5. Statistical Analysis

The continuous variables were presented as means ± standard deviations or as medians with interquartile ranges (IQRs), depending on their distributions. Categorical variables were expressed as percentages. A comparison of demographical and clinical characteristics between the patients in both groups was performed using the chi-squared test for the categorical variables and independent *t*-tests or Mann–Whitney tests, as appropriate, for the continues variables. A *p*-value of < 0.05 was considered as statistically significant. All the statistical analyses were conducted using International Business Machines Corporation’s (IBM, New York, NY, USA) SPSS Statistics, version 24.

## 3. Results

A total of 18 patients who underwent left pancreatectomy with the Da Vinci XI surgical platform were included in this study. The patients were divided into two groups: patients who consecutively received Glubran^®^2 sealant with serosal patching (GSP) (*n* = 6) versus those in which Glubran^®^2 sealant was applied without serosal patching (GNSP) (*n* = 12).

Table 1 displays the distribution of the patients’ characteristics in this study. There were no differences amongst the groups in terms of age, sex, BMI, preoperative diabetes mellitus, preoperative albumin level, ASA score, and the indication for surgery. However, there was a notable difference in the smoking status (66.7% in GSP vs. 33.3% in GNSP, *p* = 0.034) between the two groups.

Table 2 highlights the perioperative characteristics of the patients. The mean operation time was significantly longer in the GSP group (4.9 h) compared to the GNSP group (3.3 h) (*p* = 0.009). There were no significant differences in the pancreas texture, intraoperative blood loss, R0 resection, number of harvested lymph nodes, or length of hospital stay between the two groups.

Regarding postoperative outcomes, Table 3 shows that the GSP group had a significantly lower incidence rate of clinically significant POPFs (grades B/C) compared to the GNSP group (*p* = 0.034). Similarly, the overall incidence rate of POPFs (all the grades) was significantly lower in the GSP group (*p* = 0.046). There were no differences amongst the groups in terms of 90-day postoperative morbidity (Clavien–Dindo grade). There was no 90-day mortality in either group.

## 4. Discussion

This study is one of the first to investigate the combined use of Glubran^®^2 sealant with serosal patching in patients undergoing robot-assisted distal pancreatectomy. Our findings indicate that this novel approach is effective in reducing the incidence rate of clinically significant POPFs (Grades B/C).

Postoperative pancreatic fistulae (POPFs) constitute a significant complication following pancreatic resection, resulting in substantial short-term and long-term morbidities and mortalities. Characterized by their high incidence rate and considerable impact on patient outcomes, POPFs are frequently cited as a major challenge in pancreatic surgery. This complication is particularly prevalent and problematic in patients exhibiting risk factors, such as an elevated body mass index (BMI) and pancreatic tissue with reduced structural integrity (friability), underscoring the critical need for improved preventative and management strategies to reduce their occurrence and mitigate their adverse effects [17,18,19,20,21].

Numerous trials have explored various technical approaches to decrease POPF rates, including stapled closure, seromuscular patching, falciform patching with fibrin glue, trans-papillary pancreatic stenting, and pancreato-enteric anastomosis [17]. However, these methods have not consistently shown significant reductions in POPF rates. In contrast, certain interventions have demonstrated effectiveness in randomized controlled trials, such as mesh staple-line reinforcement and perioperative subcutaneous pasireotide injections. However, their benefits have not been universally confirmed across studies, and they lack widespread application [7]. Ultimately, the most important strategy for reducing the POPF incidence rate is the complete closure of the remnant pancreatic stump during surgery.

A Cochrane review by Deng, Y. et al. assessed the effectiveness and safety of fibrin sealants in preventing POPFs. That review included randomized controlled trials comparing fibrin sealants (either as a glue or patches) to controls in patients undergoing pancreatic surgery. The analysis included 11 studies with 1462 participants and revealed that fibrin sealants may have little or no effect on preventing POPFs in patients undergoing distal pancreatectomy [22]. However, the use of Glubran^®^2 specifically, was not included.

In a randomized controlled trial (DISCOVER) by Hassenpflug et al., teres ligament coverage of the pancreatic resection margin following distal pancreatectomy was compared to standard closure without coverage. Although the overall incidence rate of clinically relevant POPFs did not reach statistical significance (32.9% in the control vs. 22.4% in the teres ligament; *p* = 0.20), a statistically significant protective effect of teres ligament coverage against clinically relevant POPFs was observed (*p* = 0.0146) [23].

The efficacy of combining fibrin sealants with serosal patching to reduce the incidence rate of POPFs was also evaluated. A randomized controlled trial by Carter et al. compared standard closure (stapled or sutured) to closure reinforced with a falciform patch and a fibrin sealant for pancreatic remnant management following distal pancreatectomy. The results demonstrated no statistically significant reductions in the incidence rate or severity of POPFs with the addition of the patch and sealant [24].

A novel pancreatic remnant closure technique during open distal pancreatectomy was reported by Kelemen et al. In their study on 18 consecutive cases, they used a free fascia–peritoneum graft from the internal rectus sheet to cover the pancreatic stump. They reported promising results with zero clinically relevant postoperative pancreatic fistulae [25].

To date, the role of Glubran^®^2 in preventing POPFs has not been investigated or published in the literature. In our study, we sought to shed light on the benefits of incorporating serosal patching, specifically with Glubran^®^2, in the management of the pancreatic stump during left pancreatectomy, underscoring its impact on the incidence rate of clinically significant POPFs. For the first time, we used layers of retroperitoneal tissue as a serosal patch. We hypothesized that this technique of patching might facilitate a tight sealing of the stump, and achieve better alignment of the pancreas in its original retroperitoneal anatomical position, thereby improving the physiological flow of its secretions toward the duodenum and reducing the risk of leakage from the cut edge (Figure 2).

The overall rate of clinically significant POPFs in our cohort was 33%, not significantly different than the historical rates of Grade B or C POPFs that have been reported to range from 5% to 40% [26,27,28,29,30,31].

However, all the clinically significant POPFs were seen in the control group. The GSP group (Glubran^®^2 sealant with serosal patching) experienced a significant reduction in clinically significant POPFs (0%) compared to the GNSP group (Glubran^®^2 sealant without serosal patching) (50%), with a *p*-value of 0.034. Furthermore, the overall incidence rate of POPFs (all the grades) was also significantly lower in the GSP group (0.17 vs. 0.67) (*p* = 0.046). These findings support our hypothesis that the adhesive properties of Glubran^®^2, when combined with serosal patching, can effectively reinforce the pancreatic stump and mitigate the risk of leakage from the surgical site. Notably, these promising results were even achieved in patients with factors that might increase the risk for developing POPFs. Although their demographics were similar, the GSP group had a higher prevalence of smokers (*p* = 0.034) and a significantly longer operation time (*p* = 0.009).

The increased operation time in the GSP group was not attributable to the application of serosal patching, which ranged between 3 and 5 min, as determined by a review of our surgical videos. The primary reason for the increased duration was that the serosal patching was specifically incorporated in cases with challenging dissections, as well as in surgeries that were already prolonged, as a strategy to ensure optimal outcomes for these complex cases.

There are limitations to this study, which merit consideration. The retrospective nature of this study and the inherent limitations of a single-center design resulted in a small sample size, which limits its statistical power. Although these findings suggest a promising trend toward reduced POPFs, larger, multicenter prospective studies are warranted to further substantiate our results and provide more robust evidence on the impact of the application of serosal patching with Glubran^®^2 to the pancreatic stump on reducing postoperative pancreatic fistulae after robot-assisted left pancreatectomy.

## 5. Conclusions

Incorporating serosal patching along with Glubran^®^2 sealant in the management of the pancreatic stump following left pancreatectomy proved to be safe and feasible on the first attempt. This technique demonstrated promising results in reducing the incidence rate of clinically significant POPFs. These findings emphasize the importance of further research with larger sample sizes to confirm the observed outcomes and to explore the long-term implications of this approach for postoperative recovery and complications in patients undergoing pancreatic surgery.

## Figures and Tables

**Table 1:** Patient characteristics.

Variable	Total (18)	GSP (*n* = 6) 33.3%	GNSP (*n* = 12) 66.6%	*p*
Age (years)	67.2 + 7.9	69.5 + 5.5	66.2 + 8.9	0.42
Sex M F	10 (55.6%) 8 (44.4%)	3 (30%) 3 (37.5%)	7 (70%) 5 (62.5%)	0.73
Body mass index	26.6 ± 5.8	29.7 ± 5.1	26.6 ± 6	0.3
Preoperative diabetes mellitus Yes No	7 (39%) 11 (61%)	2 (28.6%) 4 (36.4%)	5 (71.4%) 7 (63.6%)	0.73
Smoking status Yes No	6 (33.3%) 12 (66.6%)	4 (66.7%) 2 (16.7%)	2 (33.3%) 10 (83.3%)	0.034
Preoperative albumin level	4.35 ± 0.55	4.3 ± 0.43	4.35 ± 0.62	0.97
ASA score I II–III	2 (11.1%) 16 (88.9%)	0 (0%) 6 (37.5%)	2 (100%) 10 (62.5%)	0.19
Indication for surgery Ca NET Benign	8 (44.4%) 6 (33.3%) 4 (22.2%)	3 (37.5%) 1 (16.7%) 2 (50%)	5 (62.5%) 5 (83.3%) 2 (50%)	0.52

ASA, American Society of Anesthesiology; Ca, carcinoma; NET, neuroendocrine tumor.

**Table 2:** Perioperative characteristics.

Variable	Total (18)	GSP (*n* = 6) 33.3%	GNSP (*n* = 12) 66.6%	*p*
Operation time (h)	3.8 ± 1.3	4.9 ± 1.69	3.3 ± 0.7	0.009
Pancreas texture Soft Firm	16 (88.9%) 2 (11.1%)	4 (25%) 2 (100%)	12 (75%) 0 (0%)	0.09
Estimated intraoperative blood loss (ml)	101 ± 89	105 ± 104	100 ± 85	0.9
LOS	4.7 ± 1.7	4.5 ± 1	4.75 ± 2.1	0.79
R0 resection (%)	18 (100%)	6 (100%)	12 (100%)	1
Number of harvested lymph nodes	10.3 ± 7.4	15.4 ± 6.6	8.2 ± 6.9	0.065

LOS, length of stay.

**Table 3:** Postoperative outcomes.

Variable	Total (18)	GSP (*n* = 6) 33.3%	GNSP (*n* = 12) 66.6%	*p*
Clinically significant POPFs (grades B/C) Yes No	6 (33.3%) 12 (66.7%)	0 (0%) 6 (50%)	6 (100%) 6 (50%)	0.034
POPFs (all the grades) Yes No	9 (50%) 9 (50%)	1 (11.1%) 5 (55.6%)	8 (88.9%) 4 (44.4%)	0.046
90-day postoperative morbidity (Clavien–Dindo grade) 0–I II III	13 (72.2%) 5 (27.8%) 0	4 (30.7%) 2 (40%)	9 (69.3%) 3 (60%)	0.56

POPFs, postoperative pancreatic fistulae.

## Data Availability

The data presented in this study are available on request from the corresponding author due to legal reasons.
